# A Meta-Analysis Examining the Efficacy and Predictors of Change in Mindfulness- and Self-Compassion-Based Interventions (MBSCIs) in Reducing Psychological Distress Among University Students

**DOI:** 10.3390/ejihpe16040047

**Published:** 2026-03-27

**Authors:** Cristina Galino Buen, David Martínez-Rubio, Lorena González-García, Alexandra-Elena Marin, Mª Dolores Vara, Carlos López-Pinar

**Affiliations:** 1 Department of Psychology, Faculty of Health Sciences, Universidad Europea de Valencia, 46010 Valencia, Spain; cristina.galino@universidadeuropea.es (C.G.B.); mariadolores.vara@universidadeuropea.es (M.D.V.); carloslopez.pinar@universidadeuropea.es (C.L.-P.); 2 Department of Social Psychology, Faculty of Psychology and Speech Therapy, University of Valencia, 46010 Valencia, Spain; 3 Department of Psychobiology, Faculty of Psychology and Speech Therapy, University of Valencia, 46010 Valencia, Spain; marin8@alumni.uv.es

**Keywords:** meta-analysis, mindfulness, mindfulness- and self-compassion-based interventions, psychological distress, self-compassion, university student

## Abstract

**Introduction**: University students are vulnerable to psychological distress due to the academic and social demands of this life stage. Mindfulness and self-compassion are effective and adaptable strategies in an academic environment that promote emotional regulation and psychological well-being. This study aims to conduct a systematic review and meta-analysis to evaluate the combined impact of mindfulness- and self-compassion-based interventions (MBSCIs) on psychological distress. It will also analyze their role as predictors of therapeutic change, as well as the moderating influence of sociodemographic and contextual factors. **Method**: We systematically searched PubMed, Scopus and Web of Science for randomized controlled trials (RCTs) and single-group pre-post trials investigating the effect of MBSCI on anxiety, depression and stress in college students. Studies were combined using the inverse variance method in a random effects model. Additional subgroup and meta-regression analyses were performed, and risk of bias was assessed. The review was pre-registered (PROSPERO registration number: CRD420251003822). **Results**: Our review included 49 studies with a total of 5043 participants (3721 in the intervention group, and 1322 in the control group). The results provide relevant evidence on the efficacy of MBSCI in the university population, especially in reducing symptoms of stress, anxiety, and depression. The effect sizes observed were moderate-to-large for stress and small-to-moderate for anxiety and depression, supporting their clinical usefulness in university educational settings. However, these findings should be interpreted with caution, as no included study achieved low risk of bias, and heterogeneity was moderate-to-high across most outcomes. **Conclusions**: The results suggest that MBSCI could alleviate psychological distress in university students. However, these results are limited by some methodological issues (risk of bias, heterogeneity, lack of follow-ups, poor standardization). It would be advisable to integrate these practices into the university curriculum as workshops or complementary activities. Further studies are needed to confirm their effectiveness and explore sustained effects and differences according to individual characteristics.

## 1. Introduction

College students face many challenges that can impact their academic performance and mental health (J. Zhang et al., 2024). Some of these challenges include adapting to a new academic context (Cage et al., 2021), developing new social relationships (Maunder, 2018), coping with academic stress (Córdova Olivera et al., 2023), achieving independence (Worsley et al., 2021) and entering the job market (Belle et al., 2022). According to epidemiological research, one third of the college students have met the diagnostic criteria for at least one mental disorder within the last 12 months (Auerbach et al., 2018). Depression, anxiety and stress are the most common symptoms (e.g., Mason et al., 2025; Ramón-Arbués et al., 2020). A meta-analysis by Li et al. (2022) included 64 studies with over 100,000 college students and found depression and anxiety prevalence rates of 33.6% for depression and 39% for anxiety, respectively. These rates were higher among medical students, in low- and middle-income countries, and in regions such as Africa and North America. These rates increased after the onset of the COVID-19 pandemic, reaching 35% for depression and 40.7% for anxiety. After the COVID-19 outbreak, the estimated prevalence of stress was 31% (Fang et al., 2022).

Due to the high prevalence of mental health issues, health professionals, government institutions and universities must develop and implement specific interventions to address this public health concern (Amaro et al., 2025). A systematic review by Nair and Otaki (2021), that included 40 studies from several countries, identified four categories of interventions that can help reduce psychological distress in college students. These categories included interventions based on movement and mindfulness, psychoeducation or attribution of meaning; and interventions that use support elements such as online resources or animal-assisted therapy, among others. A recent global review by Huang et al. (2024), including 74 meta-analyses, found that interventions based on exercise, cognitive behavioral therapy, mindfulness-based interventions, as well as other interventions such as acceptance and commitment therapy, effectively promote mental health among higher education students. In this sense, mindfulness-based interventions have emerged as one of the most promising approaches, because of their efficacy and their ability to be implemented in university settings (González-Martín et al., 2023). Mindfulness- and self-compassion-based interventions are growing in prominence in the scientific literature by combining contemplative practices with contemporary therapeutic approaches that foster the development of emotional self-regulation skills in college students (Ferrari et al., 2019; D. Zhang et al., 2021).

Mindfulness is defined as “a process of regulating attention in order to bring a quality of nonelaborative awareness to current experience and a quality of relating to one’s experience within an orientation of curiosity, experiential openness, and acceptance” (Bishop et al., 2004, p. 234). Mindfulness practices can be classified as formal (e.g., body scanning or breathing), or informal (e.g., mindfulness in everyday life). The main purpose of these practices is to develop and strengthen mindfulness in the present moment (D. Zhang et al., 2021). Two of the most widely used mindfulness-based intervention programs are mindfulness-based stress reduction (MBSR; Kabat-Zinn, 2003) and mindfulness-based cognitive therapy (MBCT; Segal et al., 2004; Teasdale et al., 2000).

Moreover, compassion is defined as “being moved by another’s suffering and wanting to help” (Lazarus, 1991, p. 289). Self-compassion, in turn, is defined as “being open to and moved by one’s own suffering, experiencing feelings of caring and kindness toward oneself, taking an understanding, nonjudgmental attitude toward one’s inadequacies and failures, and recognizing that one’s experience is part of the common human experience” (Neff, 2003, p. 224). The most widely used interventions for cultivating compassion and self-compassion are compassion-focused therapy (CFT; Gilbert, 2014), mindful self-compassion (MSC; Neff & Germer, 2013), loving-kindness meditation (LKM) and compassion meditation (CM; Wallmark et al., 2013).

Over the past five years, several systematic reviews and meta-analyses have been conducted to synthesize the accumulated evidence on mindfulness- and self-compassion-based interventions in the university setting. Dawson et al. (2020) included 51 RCTs that evaluated mindfulness interventions such as MBSR, MBCT, and brief mindfulness programs. They found small-to-moderate effect sizes (ESs) for reducing distress, anxiety, and depression when compared to passive controls, and small ES for improving distress and anxiety, though not depression, when compared with active controls. Similarly, Zuo et al. (2023) found slightly larger ES for reducing symptoms of depression, anxiety, and stress compared with control groups (e.g., routine healthcare, waitlist) in their review of 11 RCTs. In addition, Alrashdi et al. (2024), in a review of 26 studies, assessed the efficacy of mindfulness-based online interventions and found small to moderate ES in reducing depression, anxiety, and stress compared to passive control groups, but not to active controls. Finally, Póka et al. (2024), who included 17 RCTs, assessed the efficacy of self-compassion-based interventions (many of which were based on writing exercises) and found small ES in reducing negative affect and increasing positive affect. They found more pronounced effects when comparing self-compassion-based interventions with passive control groups than with active ones.

Empirical evidence has shown a strong correlation between mindfulness and self-compassion, suggesting that both psychological processes are closely linked and can reinforce each other (Baer et al., 2012). Specifically, it has been proposed that mindfulness facilitates the recognition of internal suffering without over-identification or reactivity, thus creating the conditions necessary to respond with kindness and shared humanity (Neff, 2023). Meanwhile, self-compassion generates a sense of emotional security that promotes open, non-avoidant observation of the experience, strengthening the attentional component of mindfulness (N. S. Schutte & Malouff, 2018). A recent meta-analysis by N. Schutte and Malouff (2025) confirmed this bidirectional relationship, showing a strong relationship between high levels of mindfulness and self-compassion (*r* = 0.53). Furthermore, although both constructs are associated with better mental health and greater well-being (Goldberg et al., 2022; Neff, 2023), they exhibit differential patterns of relationship with specific psychological variables. For example, in a large sample of the general population, self-compassion explained additional variance in depressive symptoms beyond mindfulness, while mindfulness explained unique variance in positive affect (López et al., 2016). These results highlight the specific utility of each process. Therefore, an integrated approach could provide additional benefits compared to practicing each skill in isolation. “Pure” mindfulness offers attentional regulation and cognitive disidentification, while self-compassion adds an emotional quality of care that protects against self-criticism and distress. In fact, therapeutic programs such as MSC (Neff & Germer, 2013) or interventions such as MBCT (Segal et al., 2004; Teasdale et al., 2000) incorporate both components due to their synergistic potential. Overall, the available evidence suggests that the simultaneous development of mindfulness and self-compassion could optimize therapeutic benefits and help minimize psychological distress.

In line with this approach, the recent literature has emphasized the importance of identifying the mechanisms of change in psychological interventions. This allows us to understand their effectiveness and the processes through which they generate therapeutic effects. Consistent with this idea, Collado-Navarro et al. (2021) examined the roles of mindfulness and self-compassion as mediators. They found that both mediated the effect of the MBSR program on psychological distress. However, only self-compassion explained the effect of Attachment-Based Compassion Therapy (ABCT). Similarly, López-del-Hoyo et al. (2022) found that self-kindness is key to reducing psychological distress in ABCT, while common humanity and mindfulness play relevant roles in MBSR. Furthermore, Basher (2022) demonstrated that mindfulness and self-compassion significantly predict psychological well-being. Therefore, it is crucial to further investigate these variables as potential mechanisms underlying the benefits of mindfulness- and self-compassion-based interventions.

### The Present Study

To date, systematic reviews and meta-analyses have examined the effects of mindfulness-based interventions and those focused on self-compassion separately, assessing their individual effects on the mental health of college students. In this work, we define mindfulness- and self-compassion-based interventions (MBSCIs) as interventions that intentionally and systematically incorporate core mindfulness components (e.g., present-moment awareness, non-reactivity) together with essential self-compassion elements (e.g., self-kindness, common humanity) as active therapeutic ingredients, rather than referring to these constructs only in a peripheral or incidental way. Thus, the present study aims to conduct a systematic review and meta-analysis to evaluate the efficacy of MBSCIs on psychological distress (in terms of depression, anxiety, and stress) and to analyze the role of mindfulness and self-compassion as predictors of therapeutic change process in college students. In addition, we aim to explore the potential moderator effect of several characteristics of the study design (e.g., type of comparison group), intervention (e.g., type of treatment, treatment provider, treatment delivery, and treatment format) and sample (e.g., diagnosis, mean age, and sex).

## 2. Method

This study did not receive specific funding from public, commercial, or nonprofit sources. The review followed the PRISMA guidelines for systematic reviews and meta-analyses (Page et al., 2021) with the PRISMA checklist available at this https://osf.io/6ev52/overview?view_only=04d04914342c4249aa768ff9453c3819 (accessed on 18 March 2026). In addition, this review has been pre-registered in PROSPERO (CRD420251003822). The results of the coding process (with the full codebook), the fully extracted dataset, and the R code used for the analysis can be consulted in https://osf.io/6ev52/overview?view_only=04d04914342c4249aa768ff9453c3819 (accessed on 18 March 2026).

### 2.1. Eligibility Criteria

Studies were selected using the PICOS framework:

- **Population**: College students actively enrolled at the time of the study, with no restrictions on gender or age.
- **Intervention**: MBSCI was considered within a broader classification of intervention types, which included programs based exclusively on mindfulness and compassion practices; interventions delivered within established frameworks such as MBCT or MBSR, both of which integrate self-compassion components; and other approaches based on mindfulness or self-compassion that did not fit these established models. All delivery formats were eligible: group or individual, face-to-face or online.
- **Comparison**: Active control conditions (including interventions that do not include mindfulness and self-compassion exercises), usual care, or waitlist control groups.
- **Outcomes**: Emotional distress symptoms (stress, anxiety, or depression) were measured using validated self-report or clinician-administered instruments. Process variables (mindfulness and self-compassion) were also extracted when available.
- **Study Designs**: Study designs were classified as RCTs (random allocation to conditions), non-randomized controlled trials (control group present but no random allocation), or single-arm pre-post studies (no control group, within-group change only).
- **Others**: Only peer-reviewed articles published in English or Spanish were included.

### 2.2. Information Sources and Search Strategy

A systematic search was conducted in PubMed, Scopus, and Web of Science from inception to 12 March 2025, with no date restrictions. The search strategy combined three concept blocks using Boolean operators: (1) mindfulness-based interventions (e.g., MBSR, MBCT, MBRP, mindfulness meditation); (2) self-compassion-based interventions (e.g., CFT, loving-kindness meditation, self-compassion); and (3) university or college students (including undergraduate and graduate populations). Search terms were applied to titles, abstracts, and keywords. The full search strategy is available in the https://osf.io/6ev52/overview?view_only=04d04914342c4249aa768ff9453c3819 (accessed on 18 March 2026).

Additionally, backward citation searching (reference list screening) and forward citation searching (articles citing included studies) were performed to identify additional eligible studies.

### 2.3. Selection Process

Study selection was conducted using the Rayyan (Ouzzani et al., 2016) for screening and duplicate removal. The first author conducted initial screening based on titles and abstracts. Subsequently, two independent reviewers screened the full texts of potentially eligible studies against inclusion criteria. Inter-rater agreement was excellent (*κ* = 0.86). Disagreements were resolved through discussion or consultation with a third reviewer.

### 2.4. Data Collection and Data Items

Two independent reviewers extracted data from each included study using a standardized codebook. The following information was collected: (a) study characteristics (author, year, country); (b) sample characteristics (sample size, mean age, percentage of females, diagnostic status); (c) intervention characteristics (intervention type, delivery format, duration, number of sessions); (d) comparison condition; and (e) outcome data for calculating standardized mean differences (sample size, means, and standard deviations at pre- and post-intervention for each outcome). Disagreements were resolved through consensus discussion.

### 2.5. Assessment of Risk of Bias

The Cochrane Risk of Bias Tool 2 (J. Sterne et al., 2019) was used to assess risk of bias in RCTs. Non-randomized controlled studies were assessed with Cochrane’s ROBINS-I tool (J. A. C. Sterne et al., 2016). Single-arm pre-post studies were evaluated using the National Institutes of Health Quality Assessment Tool for Before-After (Pre-Post) Studies with No Control Group (National Heart Institute, 2013). Publication bias was evaluated by visual inspection of symmetry in funnel plots, the trim-and-fill method (Duval & Tweedie, 2000), and the Egger test (Egger et al., 1997).

### 2.6. Data Analysis

Effect sizes were calculated as standardized mean differences (SMD) using Morris’s (2008) equation for between-group comparisons in RCTs. For within-group analyses, SMDs were computed for intervention groups from RCTs and single-arm studies, calculated using Morris’s (2000) formula. These formulas incorporate a correction factor for small sample sizes. For estimating the variances of both effect size indices, the Pearson correlation coefficient between the pretest and follow-up measures must be available. As this figure was not reported in the studies, a value of 0.70 was assumed for *r*, as recommended by Rosenthal (1991). Sensitivity analysis with *r* values of 0.50 and 0.90 was also conducted. A database was created containing pre-calculated SMDs and standard errors for each study and outcome.

Random-effects models were fitted using the restricted maximum likelihood method, with 95% confidence intervals adjusted using the Knapp–Hartung correction. Separate meta-analyses were performed for: (a) between-group comparisons and (b) within-group changes. Heterogeneity was assessed using *I*^2^ statistics.

Primary outcomes were emotional distress symptoms (anxiety, depression, stress). Process variables (mindfulness and self-compassion) were analyzed as secondary outcomes.

#### Additional Analyses

Moderator subgroup analyses examined categorical variables through subgroup analyses: randomization (RCTs vs. non-randomized controlled trials), diagnostic status, intervention type (mindfulness-only, compassion-only, combined), provider type, delivery format (group/individual), intervention format (face-to-face/online), comparison group type, and overall risk of bias. Between-subgroup differences were tested using Q-statistics. Additionally, a sensitivity analysis was conducted excluding studies classified as “Other” interventions to assess the robustness of the findings to the inclusion of loosely defined protocols.

Meta-regressions explored associations between effect sizes and continuous moderators (mean age, proportion of females). Additional meta-regressions tested whether changes in process variables (mindfulness and self-compassion) predicted improvements in primary outcomes.

To account for the potential dependency introduced by studies contributing multiple effect sizes, robustness analyses were conducted using Robust Variance Estimation (RVE) with small-sample correction (Hedges et al., 2010). Results are reported in Supplementary Table S3.

All the analyses were performed using the *meta* package (Balduzzi et al., 2019) in R (R Core Team, 2025). The R code and database are available at this https://osf.io/6ev52/overview?view_only=04d04914342c4249aa768ff9453c3819 (accessed on 18 March 2026).

## 3. Results

**Figure 1:**
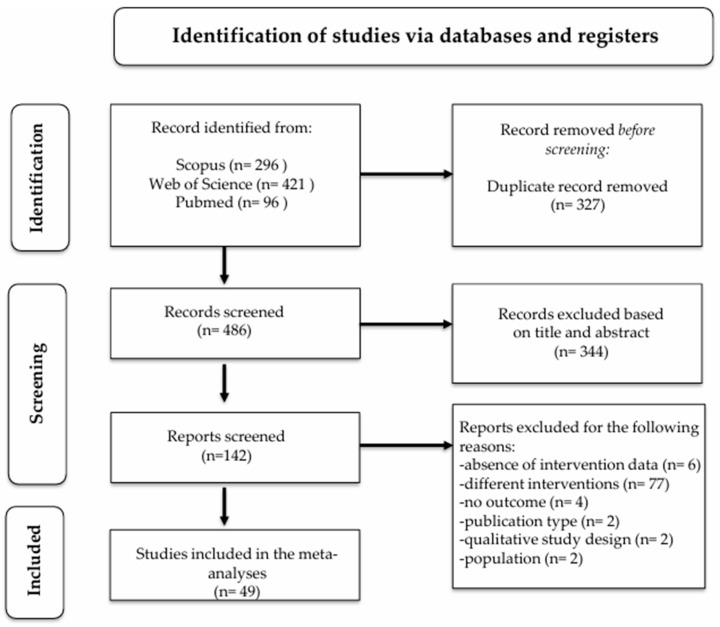
PRISMA flow diagram.

We finally identified 49 studies that met the eligibility criteria (see Figure 1 and Table 1). For the MBSCI intervention group, a total of 3721 participants were included at baseline, 3344 post-treatment, and 1140 at follow-up. A total of 27 were RCTs, nine were non-randomized controlled trials, while 13 were uncontrolled pre-post studies. Only 20% (*n* = 10) included a follow-up assessment, which ranged from 3 to 12 months (mean = 5.6 months). Most studies used generic non-manualized mindfulness and self-compassion-focused interventions (40%), followed by other protocols (32%), MBSR-based interventions (8%), and MBCT-based interventions (4%). Only 23% of the RCTs compared the intervention with an active control group. The mean age of the included sample was 22.3 years, and 76.47% of participants were women. Most interventions were delivered by a therapist (68%), were face-to-face (50%), or in a group format (56%). Seven corresponding authors were contacted to obtain missing data from the articles necessary for ES estimation. Only three responded. Two of them provided the requested data (Hindman et al., 2015; Riordan et al., 2024), and one has lost access to the data (Thomas, 2017).

Supplementary Table S1 specifies the main components of interventions classified as “Other”.

### 3.1. Risk of Bias Assessment Results

**Figure 2:**
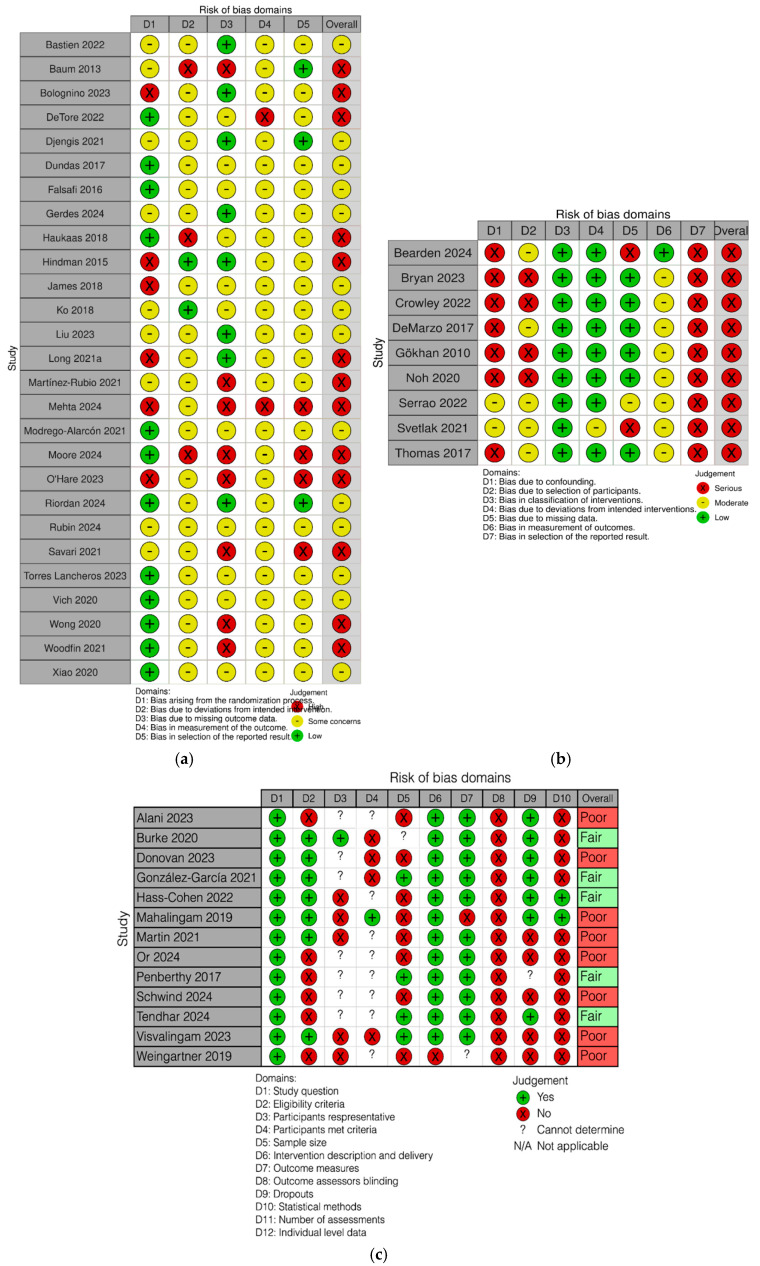
(**a**) RoB2 risk of bias summary. (**b**) ROBINS-I risk of bias summary. (**c**) NIH Quality Assessment Tool risk of bias summary.

The documents with full risk of bias results and justifications for the assessment are available at https://osf.io/6ev52/overview?view_only=04d04914342c4249aa768ff9453c3819 (accessed on 18 March 2026). The RCTs, assessed with RoB 2 (*n* = 27), were assessed with “Some concerns” (*n* = 14) or “High risk” of bias (*n* = 13; see Figure 2a). The most frequent issues identified were: (1) unclear or inadequate allocation concealment procedures (Domain 1); (2) substantial differential attrition without intention-to-treat analysis, with attrition rates ranging from 15% to 67% in some studies (Domains 2–3); (3) reliance on self-reported outcomes by unblinded participants, though this was considered acceptable under psychotherapy trial standards (Domain 4); and (4) lack of pre-registered protocols and multiple outcomes without correction for multiple comparisons (Domain 5).

For non-randomized studies evaluated with ROBINS-I (*n* = 9), all studies were rated as having “Serious risk of bias,” primarily due to self-selection into intervention groups, lack of pre-registration, high attrition rates with completers-only analyses (ranging from 43.7% to 56.3% in some studies), and reliance on self-report measures without blinding (see Figure 2b).

For single-arm pre-post studies, evaluated with the NIH Quality Assessment Tool (*n* = 13), most studies were rated as “Poor” (*n* = 8) or “Fair” (*n* = 5), with common limitations including small sample sizes, lack of pre-registration, inadequate sample size justification, high attrition rates (ranging from 12% to 40%), and self-selection bias (see Figure 2c). Overall, the three most common methodological limitations across all assessment tools were lack of pre-registered protocols, substantial attrition without appropriate handling of missing data, and self-selection bias in participant enrollment.

The publication bias assessment can be seen in Supplementary Table S2. Egger’s test was significant only for between-group stress at follow-up, within-subject depression at follow-up, and within-subject mindfulness at both post-treatment and follow-up. Further visual inspection of the funnel plot of these results was inconclusive due to the small number of studies at follow-up. Visual inspection of the funnel plot of within-subject mindfulness at post-treatment revealed a clear asymmetry. Trim-and-fill imputed additional studies for almost all outcomes. However, the adjusted SMD remained significant for all outcomes except between-groups self-compassion and within-subject anxiety, both at follow-up. Overall, the assessment of publication bias showed that the results were generally robust to this bias, with the sole exception of mindfulness after the treatment.

### 3.2. Meta-Analyses Results

**Figure 3:**
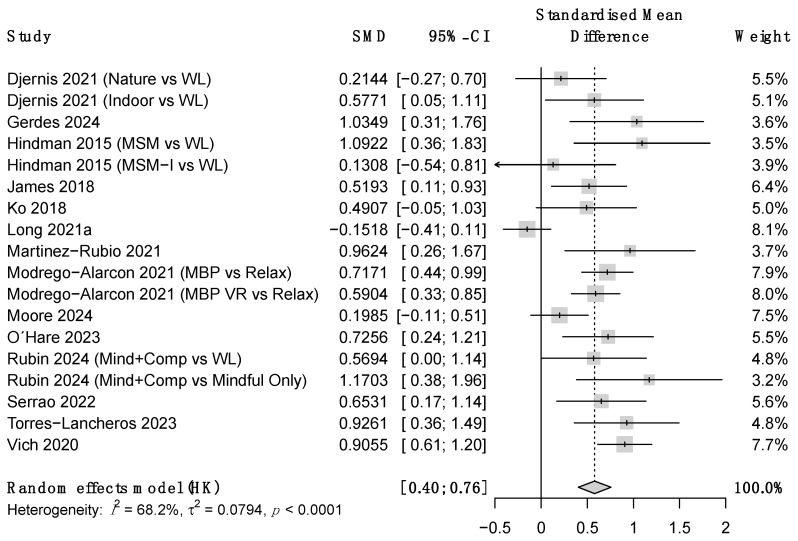
Forest plot for between-group stress at post-treatment. Note. SMD = Standardized mean differences, CI = confidence intervals.

**Figure 4:**
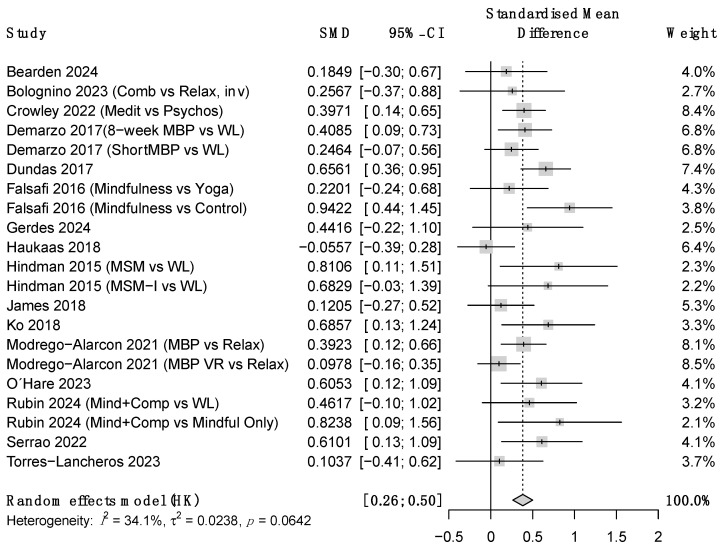
Forest plot for between-group anxiety at post-treatment. *Note.* SMD = Standardized mean differences, CI = confidence intervals.

**Figure 5:**
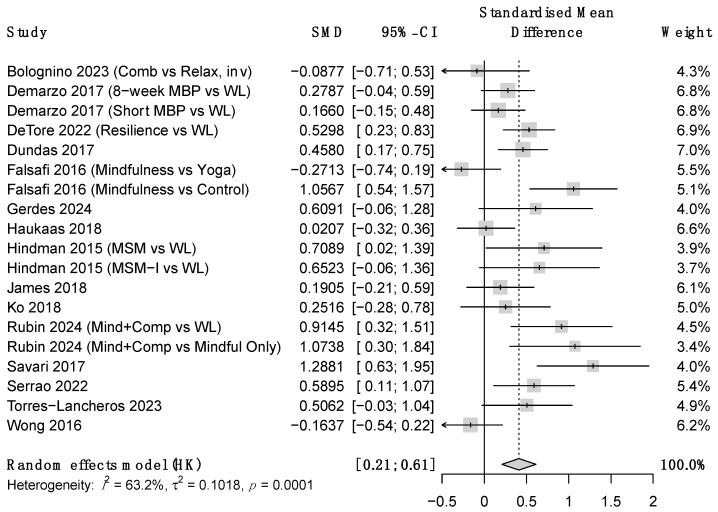
Forest plot for between-group depression at post-treatment. Note. SMD = Standardized mean differences, CI = confidence intervals.

Regarding the outcome variables, ES at post-treatment were moderate-to-large for stress and small to moderate for anxiety and depression symptoms (see Table 2 and Figure 3, Figure 4 and Figure 5). Heterogeneity was moderate-to-high for all outcomes except for between-group anxiety. At follow-up, these improvements became nonsignificant for stress and depression but increased from moderate-to-large for anxiety. However, due to the small sample size, the confidence intervals at this time point were very wide. Heterogeneity remained high at follow-up. ES estimates were very similar for between-groups comparisons and within-subject changes, though this concordance should be interpreted cautiously given that within-subject estimates from uncontrolled designs may be inflated by non-specific factors such as regression to the mean and natural symptom fluctuation. Additional analyses showed that results were that robust variance estimation (see Supplementary Table S3).

The ES estimates for process variables were also moderate to large at post-treatment, with similar between-groups and within-subject estimates. Heterogeneity was also moderate to high. At follow-up, ES remained stable and significant for all outcomes except within-subject self-compassion. Heterogeneity increased further at follow-up, and again the confidence intervals were very wide. All the results for both outcome and process variables were robust to sensitivity analysis using *r* values of 0.50 and 0.90.

### 3.3. Examining the Relationship Between the Process Variables and Psychological Distress Improvement

Effects on self-compassion significantly and positively predicted treatment improvements for all outcome variables, with the sole exception of between-group anxiety at post-treatment (see Table 3). The percentage of variance explained by the model using self-compassion ranged from 29.31% to 100% (mean = 61.78%). Models using the effect on mindfulness as a predictor also significantly and positively predicted most of the outcome variables, except for between-group and within-subject anxiety at post-treatment and between-group depression at follow-up. The percentage of variance explained ranged from 50.63% to 100% (mean = 64.16%). The extremely high *R*^2^ values may reflect overfitting.

### 3.4. Meta-Regression Results

**Figure 6:**
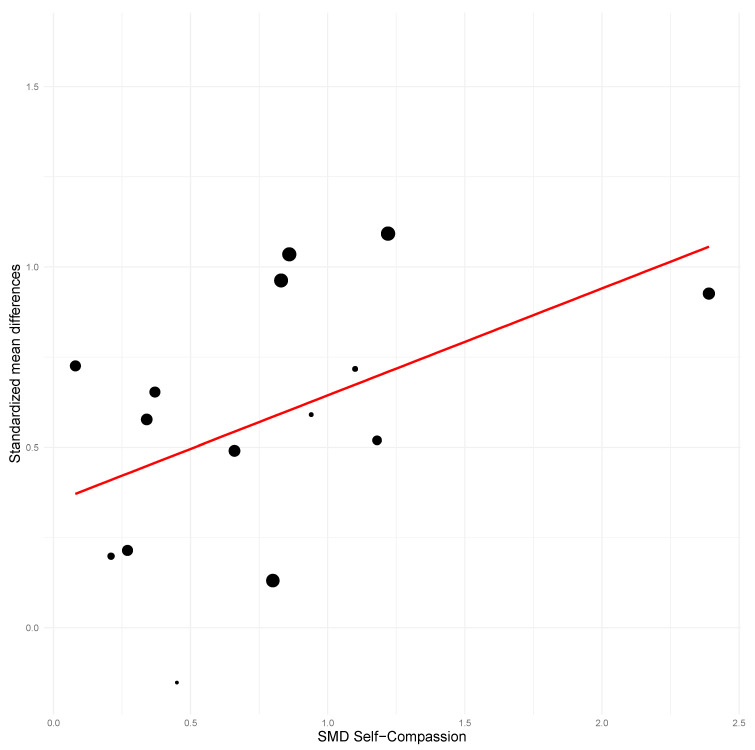
Scatter plot for between-group stress at post-treatment using self-compassion as predictor.

**Figure 7:**
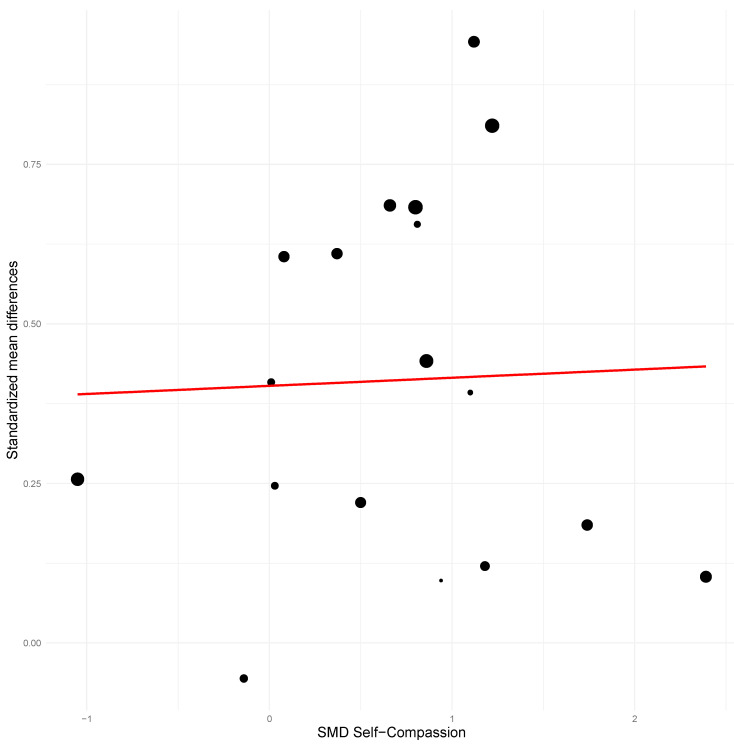
Scatter plot for between-group anxiety at post-treatment using self-compassion as predictor.

**Figure 8:**
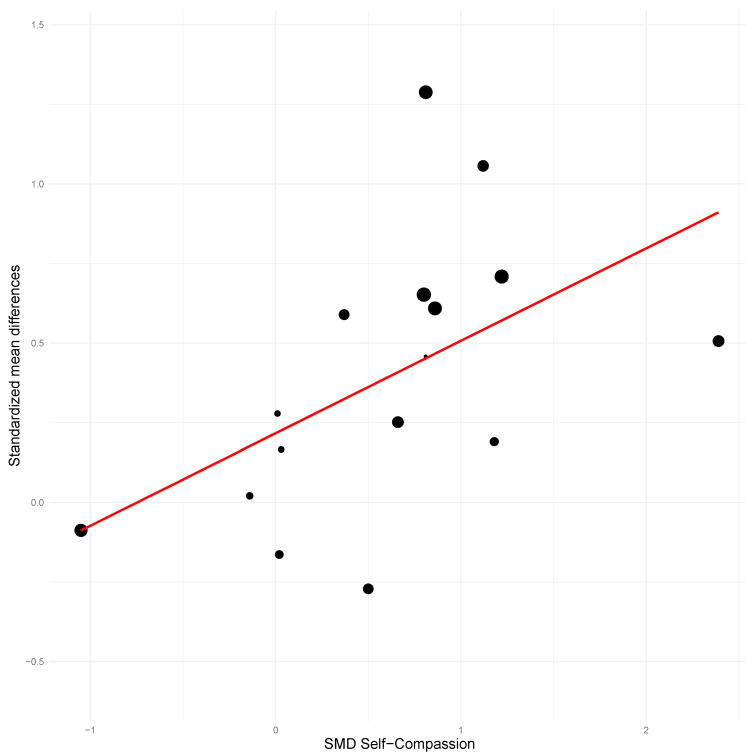
Scatter plot for between-group depression at post-treatment using self-compassion as predictor.

Mean age of participants did not predict treatment effects for most variables. Higher mean age predicted better treatment outcomes only for between-groups and within-subject depression at post-treatment (see Table 3; see Figure 6, Figure 7 and Figure 8). Age was not a significant predictor for the remaining outcomes (all *p* > 0.05). On the other hand, a higher proportion of women in the sample significantly predicted better effects for all anxiety outcomes (except for between-group anxiety at post-treatment) and for between-groups and within-subject depression at post-treatment. The coefficients of determination were higher for the models using the proportion of women as a predictor than for the models using mean age.

### 3.5. Subgroup Analyses Results

The significant subgroup analyses are shown in the Supplementary Table S3. Due to the small sample size at follow-up, only post-treatment analyses were computed.

#### 3.5.1. Randomization

No significant differences were found between RCTs and non-randomized controlled trials for any outcome (all *p* > 0.05).

#### 3.5.2. Diagnosis of the Sample

The only significant difference in treatment outcome by sample diagnosis was found in within-subject self-compassion, with Axis I diagnosed having a significantly higher effect (*Q* = 8.85; *p* = 0.001).

#### 3.5.3. Type of Intervention

Generic mindfulness and self-compassion interventions were found to be significantly more effective for within-subject anxiety (*Q* = 15.90; *p* = 0.001), and between-group depression (*Q* = 8.06; *p* = 0.04). MBCT-based interventions were significantly more effective for within-subject stress (*Q* = 26.69; *p* < 0.001) and self-compassion (*Q* = 22.32; *p* < 0.001).

The sensitivity analysis excluding “Other” interventions yielded largely consistent results. The only exception was between-group stress at follow-up, which became non-significant after the exclusion of these studies (*p* = 0.14), suggesting that this particular finding should be interpreted with caution.

#### 3.5.4. Provider of the Intervention

Several significant differences were found for this variable, but without a clear pattern. Therapist-delivered interventions were significantly more effective for between-group stress (*Q* = 7.05; *p* = 0.03), depression (*Q* = 35.30; *p* < 0.001), and self-compassion (*Q* = 11.94; *p* = 0.01). Guided interventions were significantly more effective for within-subject stress (*Q* = 19.33; *p* < 0.001). Self-delivered interventions were significantly more effective for within-subject anxiety (*Q* = 3.46; *p* = 0.001).

#### 3.5.5. Delivery of the Intervention

There is no clear pattern for this variable either. The face-to-face intervention was significantly more effective for between-groups mindfulness (*Q* = 7.81; *p* = 0.02) and self-compassion (*Q* = 13.80; *p* = 0.001). On the other hand, online-delivered interventions were significantly more effective for within-subject anxiety (*Q* = 13.80; *p* = 0.001) and combined were more effective for within-subject stress (*Q* = 20.56; *p* < 0.001).

#### 3.5.6. Format of the Intervention

Again, no clear pattern emerged for this variable, with a small superiority for group interventions. These were significantly more effective for between-group stress (*Q* = 7.02; *p* = 0.03), depression (*Q* = 35.30; *p* < 0.001), mindfulness (*Q* = 34.94; *p* < 0.001) and self-compassion (*Q* = 12.32; *p* = 0.001). Combined interventions were significantly more effective for within-subject stress (*Q* = 20.28; *p* < 0.001), while individual interventions were significantly more effective for within-subject anxiety (*Q* = 13.99; *p* = 0.001).

#### 3.5.7. Comparison Groups

No significant differences were found between comparisons against active or inactive control groups (all *p* > 0.05).

#### 3.5.8. Risk of Bias

Studies assessed with some concerns had a significantly higher ES than studies assessed with a higher risk of bias for within-subject anxiety (*Q* = 28.11, *p* < 0.001), depression (*Q* = 18.79, *p* < 0.001), and self-compassion (*Q* = 12.22, *p* = 0.01). No significant differences were found for the other outcomes (all *p* > 0.05).

## 4. Discussion

### 4.1. The Effect of MBSCI on Outcomes

This meta-analytic review aimed to quantitatively synthesize the effectiveness of MBSCI in reducing symptoms of stress, anxiety, and depression in college students, including comparisons with active and passive control groups. In addition, we examined the role of mindfulness and self-compassion as predictors of change in psychological distress. A total of 49 studies were identified integrating MBSCIs with the aim of reducing psychological distress in university students. This volume contrasts with several previous meta-analyses conducted in university populations, where practices such as mindfulness or self-compassion have been analyzed separately. Some included 51 studies on mindfulness, and others included 11, 26, and 16 studies on self-compassion (Dawson et al., 2020; Zuo et al., 2023; Alrashdi et al., 2024; Póka et al., 2024). By examining mindfulness and self-compassion together, this review integrates a wider and more representative range of research, offering a more comprehensive understanding of the impact of these contemplative practices in higher education settings.

Before discussing the findings, it is important to acknowledge the methodological limitations identified in the risk of bias assessment. None of the included studies were assessed with low risk of bias. Additionally, nine controlled studies used non-randomized designs, and only 20% of all studies included follow-up assessments. These limitations should be considered when interpreting the effect sizes and conclusions presented below. Bearing this in mind, preliminary evidence suggests that MBSCIs may be effective for reducing psychological distress among university students, with particularly notable improvements in stress symptoms. For this outcome ES reached moderate-to-large levels after the intervention, both compared to control groups and regarding within-subject change, indicating a pattern of significant clinical response. This finding is consistent with research pointing to the positive impact of mindfulness on emotional regulation and academic stress reduction (Villa Ricapa et al., 2025). Combining mindfulness with self-compassion practices could improve this effect by fostering a kinder and less reactive attitude toward difficulties and the addition of self-compassion could further modulate the threat response, promoting a sense of safety and calm (Gilbert, 2014). One possible explanation for the more pronounced effects on stress is that these symptoms, being more related to immediate coping processes, respond more readily to brief interventions; however, this mechanism has not been verified in our data, and other explanations (e.g., greater sensitivity of stress measures or floor effects for anxiety/depression in a non-clinical sample) are plausible.

In contrast, the post-treatment controlled and uncontrolled effects on anxiety and depression were more modest, with ESs ranging from small to moderate. This difference could be explained by the more chronic and multifactorial nature of these symptoms, which often require longer or more specific interventions (López-Pinar et al., 2025). These findings are consistent with those from previous reviews conducted in college students. These reported small-to-moderate effects in mindfulness-based interventions, especially when compared to passive control groups. In particular, Dawson et al. (2020), Zuo et al. (2023), Alrashdi et al. (2024), and Póka et al. (2024) identified that self-compassion-focused interventions produce small but significant effects in reducing negative affect. Thus, although MBSCI may offer benefits in these symptoms, its impact could depend on variables such as program duration, level of personal practice, or the participant’s degree of emotional involvement (Kuyken et al., 2016; Creswell, 2017; Goldberg et al., 2018).

Furthermore, at follow-up, the pattern of effects changed considerably. While anxiety symptoms showed maintained and even increased effect sizes (becoming large), improvements in stress and depression became non-significant for most comparisons. The most plausible explanation for this pattern may be the limited number of studies with follow-up assessments (only 20% of included studies, k = 4–7 per outcome), which resulted in substantially wider confidence intervals and reduced statistical power to detect effects. Additionally, the absence of booster sessions or maintenance strategies in most interventions may have contributed to the decay of effects over time, particularly for stress and depression symptoms which may require ongoing practice to sustain benefits (Kuyken et al., 2016). The variability in follow-up periods (ranging from 3 to 12 months) and potential differential attrition—where participants who maintained their mindfulness or self-compassion practice may differ from those who discontinued—could further account for the heterogeneity and instability of these estimates. Additionally, selective attrition at follow-up may have contributed to this pattern, whereby participants with poorer outcomes or lower motivation were more likely to drop out, leaving a sample biased toward those who responded better to the intervention and maintained their practice over time. These findings underscore the need for more studies incorporating longer-term follow-ups and explicit maintenance strategies to better understand the durability of MBSCI effects.

### 4.2. Relationship Between the Change in Process Variables and Psychological Distress

MBSCIs produced significant improvements in both mindfulness and self-compassion, with moderate-to-large ES at post-treatment that remained stable at follow-up. These findings may suggest that MBSCIs successfully target their intended mechanisms, fostering greater present-moment awareness and a kinder, more compassionate relationship with oneself (Neff & Germer, 2013; Bishop et al., 2004).

To examine whether improvements in process variables were associated with therapeutic changes in psychological distress, we conducted meta-regression analyses. Changes in self-compassion significantly predicted improvements across most outcomes, with a substantial portion of variance explained. The only exception was between-group anxiety at post-treatment. Similarly, changes in mindfulness significantly predicted most outcomes, showing large proportions of variance explained. However, mindfulness changes did not predict between-groups or within-subject anxiety at post-treatment, nor between-group depression at follow-up. These findings are consistent with self-compassion and mindfulness playing a role in therapeutic change in MBSCI, though these are study-level associations and do not constitute individual-level mediational evidence. However, the nature of these relationships requires further investigation through mediation studies (Collado-Navarro et al., 2021; López-del-Hoyo et al., 2022).

Nevertheless, several findings require cautious interpretation. First, the pattern for anxiety was inconsistent: while self-compassion and mindfulness predicted anxiety outcomes at follow-up and within-subject changes, they did not predict between-group anxiety at post-treatment. This suggests that mindfulness and self-compassion may be necessary but not sufficient mechanisms for addressing anxiety symptoms, or that anxiety reduction through MBSCI involves additional processes not measured in this review (Basher, 2022). Second, some *R*^2^ values were extremely high (approaching or reaching 100%), which is unusual in psychological research and likely reflects overfitting due to the small number of studies at follow-up (k = 4–10). These estimates should be interpreted with considerable caution, as they may not represent stable, generalizable relationships. Future studies with larger samples and more diverse intervention types are needed to obtain more robust estimates of the proportion of variance explained by these mechanisms.

### 4.3. Results of Moderator Analyses 

The effects of the MBSCI intervention were not consistently associated with participants’ average age, except in the case of depression, where better outcomes were associated with older age at post-treatment. By contrast, a higher proportion of women in the sample was significantly associated with improvements in anxiety and depressive symptoms at post-treatment. This variable had greater explanatory power than age. It is important to note, however, that this represents an ecological association at the study level: it indicates that studies enrolling a higher proportion of women tended to show larger effects but does not imply that women individually benefit more from the intervention. This distinction should be kept in mind to avoid ecological fallacy interpretations. These findings could inform future interventions, helping to identify which groups of college students could benefit most and at what stage of their development, as has been explored in other studies (Bluth et al., 2017). Overall, these findings suggest that the gender profile of participants may be a relevant factor to consider in the design of future MBSCIs, beyond chronological age.

Regarding diagnostic status, only one significant difference emerged: participants diagnosed with Axis I disorders showed significantly greater within-subject improvements in self-compassion compared to those without such diagnoses. This finding suggests that students with clinically significant mental health conditions may particularly benefit from the self-compassion components of MBSCIs. This is consistent with evidence indicating that self-compassion interventions can be effective in reducing symptoms among individuals with diagnosed mental disorders (Han & Kim, 2023).

Regarding study design, no significant differences in treatment effects were found between RCTs and non-randomized controlled trials across any outcome. This finding is somewhat unexpected given that randomized designs typically provide stronger internal validity. Several interpretations are possible: the effects of MBSCI may be sufficiently robust to emerge consistently across study designs, or conversely, selection bias in non-randomized studies may have inflated their estimates to a level comparable to those of RCTs. Both design types showed similar methodological limitations, as evidenced by the risk of bias assessment, and the relatively small number of non-randomized studies (*n* = 9) limited statistical power to detect differences. Nonetheless, rigorous randomized trials remain the gold standard for establishing causal inferences about intervention effects.

In terms of the type of intervention, generic MBSCI was more effective in reducing within-subject anxiety and between-group depression. This is consistent with studies supporting its positive impact on the psychological well-being of college students (e.g., Alrashdi et al., 2024; Póka et al., 2024). In contrast, MBCT-based interventions were more effective in reducing within-subject stress and increasing self-compassion. This could be explained by MBCT’s explicit focus on recognizing automatic thoughts and cultivating a compassionate attitude toward oneself. These components have been shown to be beneficial in higher education, where academic pressure and emotional distress are common (Martínez-Rubio et al., 2021; Lever Taylor et al., 2014). 

Although differences in effectiveness were observed depending on the provider, method of administration, and format of the interventions, no clear and consistent pattern emerged. Therapist-led interventions were more effective in reducing between-group stress, depression, and lack of self-compassion. In contrast, guided and self-administered interventions were more effective in reducing within-subject stress and anxiety, respectively. Face-to-face interventions were the most effective for between-group mindfulness and self-compassion, online interventions for within-subject anxiety, and combined interventions for within-subject stress. Group interventions showed some advantages in terms of between-group stress, depression, mindfulness, and self-compassion, while combined interventions were most effective for within-subject stress and individual interventions for within-subject anxiety. These results highlight the importance of tailoring interventions to participant characteristics (Harnas et al., 2024). Importantly, no significant differences were found when comparing MBSCI to active or inactive control groups. This finding suggests that MBSCI may have specific therapeutic effects attributable to its core components (mindfulness and self-compassion practices), rather than being driven primarily by non-specific factors such as therapist attention, group support, or expectancy effects.

Finally, risk of bias ratings influenced ES for some outcomes: studies with some concerns showed significantly larger effects than those with higher risk for within-subject anxiety, depression, and self-compassion, though this pattern did not emerge consistently across all outcomes. Importantly, even some concerns reflect meaningful methodological limitations, and no studies achieved low risk ratings. Therefore, while methodological quality may influence effect estimates, we cannot determine whether effects would differ with truly rigorous studies. This underscores the need for higher-quality trials with pre-registration, adequate allocation concealment, intention-to-treat analyses, and minimal attrition.

### 4.4. Limitations

This study has several methodological limitations that should be considered when interpreting the results. First, there is considerable heterogeneity among the studies included, both in terms of design and in the characteristics of the interventions and populations evaluated. This variability is reflected in the range of effect sizes and the dispersion of results, which hinders comparative synthesis and limits the generalizability of conclusions.

Significant methodological weaknesses were identified across included studies. No study achieved “low risk” of bias. Common issues in RCTs included inadequate allocation concealment, substantial attrition without intention-to-treat analysis, lack of pre-registration, and multiple untested outcomes. Additionally, several studies used non-randomized designs, limiting causal inference. The minority of studies included follow-up assessments, restricting understanding of long-term effects. Sample sizes were generally moderate, potentially affecting statistical power, particularly for subgroup analyses. All studies relied on self-reported outcomes from unblinded participants, though this is inherent to psychotherapy research. These limitations substantially constrain the strength of conclusions.

The diversity of intervention types is another relevant factor. Although 40% of the studies used generic MBSCI, only a small percentage were based on standardized protocols such as MBSR (8%) or MBCT (4%). This lack of methodological uniformity may influence the consistency of the observed effects and the replicability of the results. Furthermore, only 20% of the studies included follow-up assessments, which limits our understanding of the sustained impact of interventions in the medium and long term.

Subgroup analyses were limited to post-intervention data due to the small sample size in the follow-up evaluations. This restriction prevents in-depth exploration of the differential effects of the different types of intervention (face-to-face, online, combined) and their interaction with moderating variables such as gender, academic year, or symptom level.

In terms of sociodemographic characteristics, the sample was composed mainly of people who identified as female (76.47%) and with an average age of 22.3 years, which limits the possibility of extrapolating the results to people of other genders or significantly younger or older ages.

Moreover, although self-compassion and mindfulness were identified as a significant predictor of variability in outcomes, mindfulness did not predict post-treatment anxiety or follow-up depression. The limited inclusion of process measures in the studies limits the possibility of establishing robust explanatory mechanisms, and the lack of mediational and longitudinal analyses prevents a clear determination of the modulatory role of both mindfulness and self-compassion in the evolution of symptoms.

The inclusion of non-randomized and uncontrolled designs alongside RCTs limits causal inference. In particular, within-subject effect size estimates may be inflated by confounds such as regression to the mean, natural symptom fluctuation, and non-specific effects. These estimates should therefore be interpreted with caution and not used to draw conclusions about treatment efficacy. Nonetheless, between-group and within-subject analyses were conducted separately, and sensitivity analyses revealed no significant differences in effect sizes between randomized and non-randomized controlled studies, suggesting that design heterogeneity did not substantially bias the primary efficacy estimates.

Restricting the search to English and Spanish publications may have introduced language bias, potentially excluding relevant studies published in other languages.

The search was limited to PubMed, Scopus, and Web of Science; the omission of PsycINFO and the Cochrane Central Register of Controlled Trials may have introduced search bias and cannot be ruled out as a source of missed eligible studies.

Finally, the inconsistent reporting of intervention dose across studies (e.g., total hours of practice, session length, and home practice frequency) prevented examination of dose–response relationships, which may be an important moderator of treatment outcomes.

### 4.5. Implications for Clinical Practice

The results of this study provide a solid empirical basis for guiding the development of psychoeducational interventions aimed at the university population. First, it highlights the importance of incorporating MBSCIs into the academic setting, given their potential effectiveness in improving psychological well-being and emotional self-regulation in the face of academic stress.

From an institutional perspective, it is recommended that these practices be integrated into the university curriculum, either as complementary activities, extracurricular workshops, or cross-disciplinary training modules. This strategy would allow for systematic and accessible implementation, promoting the normalization of self-care practices among students.

It also highlights the need to train teaching and support staff in skills related to self-compassion and mindfulness, with the aim of creating more empathetic, inclusive, and emotionally secure educational environments. This training could contribute to improving the quality of studies and to the early detection of indicators of psychological distress in students.

There is a need to adapt interventions to the individual characteristics of students, considering variables such as gender, academic level, area of study, and the presence of symptoms. This personalization would facilitate greater adherence to programs and optimize results. Further studies are needed to confirm these findings on the efficacy of third-generation behavioral psychotherapies in treating anxiety symptoms (Bennett et al., 2021; Bijulakshmi & Kumar, 2025).

## 5. Conclusions

This meta-analytic review focused on quantitatively synthesizing the effectiveness of MBSCI on psychological distress in university students, considering symptoms of depression, anxiety, and stress, as well as the relationship of self-compassion and mindfulness with change in output variables.

The results provide preliminary evidence suggesting the potential usefulness of MBSCI in university settings. Moderate-to-large effect sizes were observed for stress reduction, while effects on anxiety and depression were more modest (small-to-moderate). However, these findings must be interpreted with considerable caution given the significant risk of bias identified in the included studies, the fact that several used non-randomized designs, and that only a small minority included follow-up assessments. Additionally, improvements in mindfulness and self-compassion significantly predicted symptom reduction, suggesting they may be associated with therapeutic change, though these findings do not establish a mechanistic role. Nevertheless, the correlational nature of these analyses and extreme *R*^2^ values in some cases limit causal interpretation.

Additional significant methodological limitations constrain the strength of conclusions. High heterogeneity across studies, limited longitudinal data, gender imbalance (76% women), lack of standardization in intervention protocols, and absence of rigorous randomized trials restrict generalizability and prevent identification of consistent mechanisms. Therefore, while MBSCI shows promise, the evidence base remains preliminary.

Despite these limitations, MBSCI may offer a feasible approach for supporting student mental health in higher education settings, particularly for stress management. Integration into university wellness programs, curriculum-based workshops, or staff training could be considered, though implementation should be accompanied by rigorous evaluation to determine effectiveness in specific contexts.

Future research should prioritize methodologically rigorous trials with pre-registration, adequate randomization and allocation concealment, intention-to-treat analyses, longer follow-up periods, and more diverse samples. Mediation studies are needed to clarify the mechanisms through which MBSCI produces effects. This work provides a foundation for understanding MBSCIs’ potential in higher education while highlighting the substantial methodological improvements needed to establish robust, generalizable evidence for these interventions.

## Figures and Tables

**Table 1:** Main characteristics of the included studies.

Study	Country	Design	N Total	Base-Line N (Intervention)	Diagnosis of the Participants	Mean Age	% Female	Intervention	Length (Sessions/Weeks)	Application	Delivery	Format	Comparison
Alani et al. (2023)	United Arab Emirates	Single-arm trial	39	39	No diagnosis	22.3	74.36	Mindfulness + self-compassion	1 week	Self-delivered	N/A	Individual	-
Bastien et al. (2022)	Canada	RCT	217	142	No diagnosis	20.44	78.8	Other	3 sessions/4 weeks	Self-delivered	Online	Individual	Both
Baum and Rude (2013)	U.S.A.	RCT	218	145	No diagnosis	21	72.44	Mindfulness + self-compassion	3 sessions/1 week	Self-delivered	Online	Individual	Both
Bearden et al. (2024)	Canada	Non-randomized	45	15	No diagnosis	23.48	78.89	MBSR	8 weeks (weekly classes)	Therapist	Face-to-face	Group	Inactive
Bolognino et al. (2023)	U.S.A.	RCT	52	38	No diagnosis	20	75	Mindfulness + self-compassion	14 sessions (daily)/2 weeks	Self-delivered	Online	Individual	Active
Bryan et al. (2023)	U.S.A.	Non-randomized	42	21	No diagnosis	20.5	71	Mindfulness + self-compassion	16 weeks	Therapist	Online	Group	Inactive
Burke et al. (2020)	U.S.A.	Single-arm trial	63	63	No diagnosis	19.34	65	Other	4 sessions/4 weeks	Therapist	Face-to-face	Group	-
Crowley et al. (2022)	U.S.A.	Non-randomized	147	74	No diagnosis	21.1	60.45	Mindfulness + self-compassion	15 weeks	Therapist	Face-to-face	Group	Active
Demarzo et al. (2017)	Spain	Non-randomized	141	94	No diagnosis	21.52	68.9	MBSR	8 sessions/8 weeks and an abbreviated version: 4 sessions/4 weeks	Therapist	Face-to-face	Group	Both
DeTore et al. (2023)	U.S.A.	RCT	107	54	No diagnosis	N/A	71.03	Other	4 sessions/4 weeks	Therapist	Face-to-face	Group	Inactive
Djernis et al. (2021)	Denmark	RCT	60	42	No diagnosis	30.6	86.7	MBSR	5 consecutive-day retreat	Therapist	Face-to-face	Group	Both
Donovan et al. (2023)	U.S.A.	Single-arm trial	25	25	No diagnosis	20.35	92	Mindfulness + self-compassion	8 sessions/8 weeks	Therapist	Face-to-face	Group	-
Dundas et al. (2017)	Denmark	RCT	158	69	No diagnosis	25	85	Mindfulness + self-compassion	3 sessions (90 min)/2 weeks	Therapist	Face-to-face	Group	Inactive
Falsafi (2016)	U.S.A.	RCT	90	60	Axis I	22.1	86.4	Mindfulness + self-compassion	8 sessions/8 weeks (75 min/week)	Therapist	Face-to-face	Group	Both
Gerdes & Gordon (2025)	U.S.A.	RCT	24	13	No diagnosis	22	79	Other	6 sessions/6 weeks (1 h/week)	Therapist	Face-to-face	Group	Inactive
Gökhan et al. (2010)	U.S.A.	Non-randomized	42	22	No diagnosis	24.25	78.57	MBSR	12 weeks (within a 14-week course)	N/A	Face-to-face	Group	Active
González-García et al. (2021)	Spain	Single-arm trial	66	66	No diagnosis	19.83	86.36	Mindfulness + self-compassion	16 days (intensive intervention)	Self-delivered	Online	Individual	-
Hass-Cohen et al. (2023)	U.S.A.	Single-arm trial	18	18	No diagnosis	32.22	83	Other	10 sessions/2 weekends	Therapist	Face-to-face	Group	-
Haukaas et al. (2018)	Norway	RCT	81	81	No diagnosis	22.9	75.3	Mindfulness + self-compassion	3 sessions	Therapist	Face-to-face	Group	Active
Hindman et al. (2015)	U.S.A.	RCT	34	24	No diagnosis	22.35	88.2	Mindfulness + self-compassion	4–8 sessions	Therapist	Face-to-face	Group	Both
James and Rimes (2018)	United Kingdom	RCT	60	28	Axis I	N/A	81.65	MBCT	8 sessions/8 weeks	Therapist	Face-to-face	Group	Active
Ko et al. (2018)	U.S.A.	RCT	41	20	No diagnosis	19.78	66	Other	1 semester (12–14 weeks)	N/A	Face-to-face	Group	Inactive
Liu et al. (2023)	China	RCT	74	37	No diagnosis	17.73	60.8	Other	8 weeks (weekly classes)	Self-delivered	Online	Individual	Inactive
Long et al. (2021)	U.S.A.	RCT	208	208	No diagnosis	N/A	73	Other	6 sessions/6 weeks (90 min/week)	Therapist	Face-to-face	Group	Inactive
Mahalingam and Rabelo (2019)	U.S.A.	Single-arm trial	24	24	No diagnosis	21.92	70.83	Mindfulness + self-compassion	14 classes (2 per week/7 weeks)	Therapist	Face-to-face	Group	-
Martin et al. (2021)	U.S.A.	Single-arm trial	39	39	No diagnosis	N/A	N/A	Mindfulness + self-compassion	1 single workshop	Therapist	Face-to-face	Group	-
Martínez-Rubio et al. (2021)	Spain	RCT	30	15	No diagnosis	22.29	83	MBCT	6 sessions/6 weeks	Therapist	Face-to-face	Group	Inactive
Mehta et al. (2024)	U.S.A.	RCT	17	11	No diagnosis	N/A	N/A	Other	8 sessions	Therapist	Face-to-face	Group	Active
Modrego-Alarcón et al. (2021)	Spain	RCT	280	150	No diagnosis	22.25	78.9	Mindfulness + self-compassion	6 weeks (weekly sessions)	Guided	Combined	Combined	Active
Moore et al. (2024)	Australia	RCT	114	57	No diagnosis	25.25	74.4	Mindfulness + self-compassion	8 weeks	Self-delivered	Online	Individual	Inactive
Noh and Cho (2020)	South Korea	Non-randomized	38	18	No diagnosis	21.55	57.91	Other	8 sessions	Therapist	Face-to-face	Group	Inactive
O’Hare and Gemelli (2023)	U.S.A.	RCT	52	52	No diagnosis	22.99	85.76	Mindfulness + self-compassion	10 weeks	Therapist	Face-to-face	Group	Active
Or et al. (2024)	U.S.A.	Single-arm trial	58	58	No diagnosis	N/A	87.9	Other	Brief practice: 8 min, 3–4 times/week for 2 weeks	Self-delivered	Online	Individual	-
Penberthy et al. (2017)	U.S.A.	Single-arm trial	205	205	No diagnosis	20.7	68.1	Mindfulness + self-compassion	14 weeks, weekly practices	Therapist	Face-to-face	Group	-
Riordan et al. (2024)	U.S.A.	RCT	351	351	No diagnosis	20.17	77.8	Other	2 weeks (20 min/day)	Self-delivered	Online	Individual	Active
Rubin et al. (2024)	U.S.A.	RCT	91	61	No diagnosis	27.32	60.44	Mindfulness + self-compassion	1 session, 1 h	Therapist	Online	Group	Both
Savari et al. (2021)	Iran	RCT	30	15	Axis I	24.3	100	Mindfulness + self-compassion	8 sessions/(2 per week/4 weeks)	Therapist	Face-to-face	Group	Inactive
Schwind et al. (2024)	Canada	Single-arm trial	25	25	No diagnosis	N/A	84	Mindfulness + self-compassion	3 workshops + daily practice during the semester	Therapist	Online	Group	-
Serrão et al. (2022)	Portugal	Non-randomized	44	23	No diagnosis	19.51	86.36	Mindfulness + self-compassion	12 weeks	Therapist	Face-to-face	Group	Inactive
Světlák et al. (2021)	Czech Republic	Non-randomized	692	333	No diagnosis	22.9	81.3	MBCT	8 weeks	Guided	Combined	Combined	Inactive
Tendhar et al. (2024)	U.S.A.	Single-arm trial	92	92	No diagnosis	20.39	86	Other	8 sessions	Self-delivered	Online	Individual	-
Thomas (2017)	U.S.A.	Non-randomized	99	66	No diagnosis	N/A	90	Mindfulness + self-compassion	100 min/16 weeks	Therapist	Face-to-face	Group	Active
Torres Lancheros et al. (2023)	Colombia	RCT	35	18	No diagnosis	23.25	N/A	Mindfulness + self-compassion	4 sessions (2 h per session)/4 weeks	Therapist	Online	Group	Inactive
Vich et al. (2020)	Czech Republic	RCT	128	64	No diagnosis	23.4	62.5	Other	8 sessions (2 h per sessions + 1 intensive 6 h session/8 weeks	Therapist	Face-to-face	Group	Active
Visvalingam et al. (2023)	Australia	Single-arm trial	70	70	No diagnosis	19	60	Other	Single 2 h intervention + assignments/2 weeks	Self-delivered	Online	Individual	-
Weingartner et al. (2019)	U.S.A.	Single-arm trial	45	45	No diagnosis	N/A	N/A	Other	8 weeks (weekly sessions + 2 h daily practice)	Therapist	Face-to-face	Group	-
Wong and Mak (2016)	China	RCT	112	33	No diagnosis	20.5	53.8	Mindfulness + self-compassion	3 written practices per week/1 week	Self-delivered	Online	Individual	Inactive
Woodfin et al. (2021)	Norway	RCT	221	89	No diagnosis	27	75.76	Mindfulness + self-compassion	5 sessions (4 seminars + a 4 h retreat)/3 weeks	Therapist	Face-to-face	Group	Inactive
Xiao et al. (2020)	China	RCT	99	49	No diagnosis	18	76	Mindfulness + self-compassion	11 weeks	Therapist	Face-to-face	Group	Inactive

Note. N = participants; N/A = Not Available.

**Table 2:** Standardized mean differences (SMD), 95% confidence intervals, heterogeneity analyses (*I*^2^), and risk of bias for between-groups and within-subject outcomes.

Variable		Time Point	Between-Groups Outcomes					Within-Subject Outcomes				
			*k*	*n*	SMD	95% CI	*I* ^2^	*k*	*n*	SMD	95% CI	*I* ^2^
Outcome variables	Stress	PT	18	1094	0.58	0.40 to 0.75	68.2	23	1104	0.57	0.43 to 0.71	80.7
		FU	4	514	0.60	0.04 to 1.16	81.4	4	289	0.45	−0.42 to 1.33	96.2
	Anxiety symptoms	PT	21	1315	0.38	0.26 to 0.50	34.1	27	1462	0.45	0.34 to 0.56	77.4
		FU	7	642	0.80	0.24 to 1.36	90.9	6	319	0.70	0.08 to 1.32	94.1
	Depression symptoms	PT	19	996	0.41	0.21 to 0.61	63.2	24	1082	0.38	0.26 to 0.51	73.8
		FU	6	422	0.32	−0.13 to 0.77	68.9	5	189	0.40	−0.26 to 1.06	79.5
Process variables	Mindfulness	PT	30	2156	0.57	0.43 to 0.70	69.5	38	1895	0.55	0.44 to 0.68	82.5
		FU	9	871	0.65	0.31 to 0.99	86.1	8	445	0.95	0.43 to 1.47	93.9
	Self-Compassion	PT	30	1887	0.60	0.38 to 0.82	82	43	2257	0.61	0.46 to 0.76	87.1
		FU	10	936	0.58	0.09 to 1.06	92.5	9	478	0.62	−0.07 to 1.30	96.4

Note. CI = confidence intervals; FU = follow-up; *k* = number of studies; *n* = number of participants; PT = post-treatment; SMD = standardized mean differences.

**Table 3:** Meta-regression results with the betas, standard errors, *p* values, and coefficient of determination (*R*^2^).

Outcome	Covariates															
	Percentage of Women				Age of Participants				Mindfulness				Self-Compassion			
	ß	SE	*p*	*R* ^2^	ß	SE	*p*	*R* ^2^	ß	SE	*p*	*R* ^2^	ß	SE	*p*	*R* ^2^
BG Stress (post-treatment)	−0.01	0.01	0.73	0	−0.03	0.02	0.26	15.46	**1.06**	**0.18**	**<0.001**	**93.77**	**0.32**	**0.15**	**0.06**	**29.31**
BG Stress (follow-up)	Insufficient studies															
WS Stress (post-treatment)	−0.01	0.01	0.86	0	−0.04	0.02	0.09	16.32	**0.63**	**0.17**	**0.001**	**57.75**	**0.36**	**0.10**	**0.003**	**42.75**
WS Stress (follow-up)	0.05	0.03	0.29	26.16	−0.34	0.12	0.11	71.53	**0.69**	**0.08**	**0.01**	**99.23**	**0.68**	**0.07**	**0.01**	**99.96**
BG anxiety (post-treatment)	0.01	0.01	0.54	0	0.01	0.03	0.67	0	0.22	0.19	0.27	20.19	0.01	0.10	0.89	0
BG anxiety (follow-up)	**0.06**	**0.02**	**0.04**	**54.46**	−0.02	0.56	0.96	0	**0.97**	**0.42**	**0.07**	**50.63**	**0.73**	**0.14**	**0.01**	**89.83**
WS anxiety (post-treatment)	**0.01**	**0.01**	**0.001**	**35.29**	0.04	0.03	0.18	7.5	0.10	0.09	0.28	0.29	**0.29**	**0.10**	**0.001**	**43.20**
WS anxiety (follow-up)	**0.08**	**0.02**	**0.01**	**82.97**	0.36	0.53	0.54	0	**0.85**	**0.14**	**0.01**	**94.70**	**0.61**	**0.09**	**0.01**	**94.38**
BG depression (post-treatment)	**0.01**	**0.01**	**0.18**	**13.27**	**0.10**	**0.04**	**0.01**	**28.94**	**0.62**	**0.26**	**0.04**	**61.58**	**0.28**	**0.13**	**0.04**	**41.56**
BG depression (follow-up)	0.01	0.02	0.41	0	0.04	0.27	0.87	0	0.85	0.54	0.21	32.49	**0.46**	**0.18**	**0.06**	**100**
WS depression (post-treatment)	**0.02**	**0.01**	**0.002**	**58.11**	**0.08**	**0.03**	**0.02**	**19.13**	**0.42**	**0.09**	**0.001**	**100**	**0.33**	**0.12**	**0.01**	**42.93**
WS depression (follow-up)	0.03	0.02	0.15	49.35	0.21	0.33	0.56	0	**1.20**	**0.33**	**0.06**	**95.12**	**0.46**	**0.14**	**0.05**	**95.67**

Note. Significant results are bolded, BG = between-groups, WS = within-subject.

## Data Availability

The R code and database are freely available at this https://osf.io/6ev52/overview?view_only=04d04914342c4249aa768ff9453c3819 (accessed on 18 March 2026).

## Supplementary Materials

The following supporting information can be downloaded at https://www.mdpi.com/article/10.3390/ejihpe16040047/s1, File S1: Tables S1–S3; File S2: PRISMA checklist.
