# Supplementary material for: A Meta-Analysis Examining the Efficacy and Predictors of Change in Mindfulness- and Self-Compassion-Based Interventions (MBSCIs) in Reducing Psychological Distress Among University Students
Source: Eur J Investig Health Psychol Educ. 2026 Mar 27;16(4):47. doi: 10.3390/ejihpe16040047 (PMC13115322; doi:10.3390/ejihpe16040047)
Supplement: Supplementary file 1 [file ejihpe-16-00047-s001.zip › ejihpe-4160047-supplementary File S1.pdf]

Supplementary Table S1

Characteristics of interventions classified as "other"

Study	Intervention
Bastien et al. (2022)	SCOOP (web-based resilience program): 3 brief scripted videos delivered over ~4 weeks + access for ~9 weeks to an online resource library (infographics, guided audios, podcasts) on stress, self-criticism, self-care/help-seeking, and social connection/support. Presenter was either a mental health provider or a peer (same scripted content).
Burke et al. (2020)	Transdiagnostic prevention program. The elements it incorporates are established mindfulness, conscious self-compassion, and mentalization-based interventions (skills taught via experiential exercises).
DeTore et al. (2022)	Resilience training. This program focuses on developing skills to improve resilience using materials adapted from established mindfulness, self-compassion, and mentalization interventions, and how these concepts and skills can be applied in everyday life.
Gerdes & Gordon (2024)	Wellness program, based on relaxation and mindfulness, yoga, gratitude and self-compassion, emotion regulation, goal setting and time management, nutrition and movement.
Hass-Cohen et al. (2022)	The practices included nourishing inbreaths, releasing outbreaths, warm and soothing touch, mindfulness and mindful self-compassion breaks, and appreciative rocking and walking meditations. Core meditations included: a friendly body scan, soft and friendly breathing, choiceless awareness and acceptance, loving kindness, and visual contemplation. Art therapy directives and inspirational art imagery were used as prompts. Inspirational images included art from the Prinzhorn collection and Art Brut, modern and abstract expressionism, and crafts.
Ko et al. (2018)	Seminar on compassion. It is based on different contemplative methods: mindfulness, sitting meditation, deep listening, nature observation, walking meditation, walking a labyrinth, metta (loving-kindness meditation), tonglen meditation (Tibetan Buddhism), Mother Teresa's method of seeing Christ in every person, Sufi dance and poetry, and others.
Liu et al. (2023)	Short-video app–guided loving-kindness meditation (animation-guided): guided LKM practice cultivating kindness/compassion through blessing phrases directed progressively to self, loved ones, neutral persons, difficult persons, and ultimately all beings
Long et al. (2021)	The program is based on a combination of contemplative practices and training in coping and emotional regulation skills based on cognitive-behavioral (CB).
Mehta et al. (2024)	The intervention was structured in a gradual manner: first, the meaning of compassion as a concept and as a practice was explored through shared experiences. Then, participants worked on calming the mind with mindfulness exercises. They were invited to reflect on their own emotions, personal needs, and associated behaviors. Common needs and values that promote connection with others were also discussed, as well as the barriers that hinder that connection and the role of stereotypes in interactions. Finally, the concept of shared humanity was introduced through mental exercises and its practical application in a clinical setting.
Noh & Cho (2020)	Mindful Lovingkindness Compassion Program. This program is based on progressive practices of mindfulness, emotion regulation system (within compassion training), and cultivating compassion, organized into modules that include: exploring participants' needs and expectations; introduction to meditation and the nature of the mind; training in mindfulness of breathing, sounds, bodily sensations, thoughts, and emotions; specific work with self-criticism; development of kindness, compassion, and the figure of the "compassionate nurturer"; identification of personal compassionate attributes; practices directed towards oneself, towards the self-critical part, towards loved ones, group members, and strangers; and finally, a deep reflection on the meaning of life and the integration of everything learned for use in everyday life.

Or et al. (2024)	The intervention was supported by an audio recording of loving-kindness meditation based on the script developed by Levett-Jones, which participants used to guide themselves through the mindfulness activity.
Riordan et al. (2024)	Modified version of the Healthy Minds Program (HMP) app: guided meditation practices along with brief psychoeducational content covering the science of well-being. Although the full HMP has four skill-based training modules (awareness, connection, insight, purpose), this trial used content only from the connection module (primarily compassion-based practices).
Tendhar et al. (2024)	The program is based on the Seven-Point Cause and Effect Instructions of Buddha Maitreya. Building on these teachings, the curriculum is organized into eight progressive lessons, mindfulness, common humanity, gratitude, loving-kindness, empathic concern, forgiveness, self-compassion, and compassion for others, designed to develop a deep understanding of compassion.
Vich et al (2020)	Relational Mindfulness Training (RMT) focuses primarily on the practice of relational mindfulness. The program also includes individual practice based on classic mindfulness meditations and loving-kindness meditations (LKM), which help participants become familiar with the development of mindfulness, compassion, and other related qualities in solitude.
Visvalingam et al. (2023)	The Intentional Imperfection Program (IIP) to reduce perfectionism. The learning modules are as follows: psychoeducation on perfectionism, interpersonal sensitivity psychoeducation and management skills, interpersonal hostility psychoeducation and management skills, social disconnection psychoeducation and strategies to increase social connection, and a summary of the learnt materials.
Weingartner et al. (2019)	Modeled after CCARE's Compassion Cultivation Training (CCT), adapted to medical students (incl. compassion exercises focused on patient interactions). The sessions included pedagogical instruction, guided group meditation, mindfulness training, group discussion, listening and communication exercises, and applied practices related to the weekly compassion themes.

Supplementary Table S2

Publication bias analyses results

	Between-groups outcomes					Within-subject outcomes				
	Trim and Fill					Trim and Fill				
Outcome	k	Studies imputed	Adjusted SMD	95% CI	Egger test	k	Studies imputed	Adjusted SMD	95% CI	Egger test
Stress	18	4	0.49	0.31/0.67	1.62	23	0	-	-	0.22
Stress (Follow-up)	4	-	-	-	-27.66***	4	1	0.26	-0.54/1.06	3.11
Anxiety	21	5	0.31	0.19/0.44	1.70	27	7	0.33	0.20/0.46	1.32
Anxiety (Follow-up)	7	2	0.56	0.01/1.11	0.54	6	1	0.57	-0.04/1.18	1.01
Depression	19	4	0.28	0.04/0.51	1.91	24	7	0.29	0.15/0.43	1.57
Depression (Follow-up)	6	0	-	-	1.77	5	0	-	-	4.63*
Mindfulness	30	10	0.39	0.23/0.54	2.46	38	13	0.37	0.23/0.52	3.26***
Mindfulness (Follow-up)	9	0	-	-	-0.56	8	1	0.79	0.22/1.38	2.52*
Self-compassion	30	5	0.45	0.20/0.70	1.51	43	8	0.45	0.27/0.63	1.82
Self-compassion (Follow-up)	10	1	0.47	-0.03/0.97	0.52	9	1	0.45	-0.27/1.18	1.49

Note. * = $p > 0.05$; ** = $p > 0.01$; *** = $p > 0.001$; SMD = standardized mean differences; CI = confidence intervals

Supplementary Table S3

Statistically significant subgroup analyses results

Outcome	Variable	*Q* test (*p* value)	Subgroup	k	SMD	95% CI
Between-groups stress	Provider	7.05 (.03)	Self-delivered	1	0.20	-0.11 to 0.51
			**Therapist**	**14**	**0.62**	**0.39 to 0.85**
			Guided	2	0.65	-0.15 to 1.45
	Format	7.02 (.03)	Individual	1	0.19	-0.11 to 0.51
			**Group**	**15**	**0.61**	**0.39 to 0.82**
			Combined	2	0.65	-0.15 to 1.45
Within-subject stress	Intervention	26.69 (<.001)	Mindful+Comp.	13	0.61	0.40 to 0.83
			MBRS	2	0.16	-2.07 to 2.39
			**MBCT**	**4**	**0.75**	**0.62 to 0.89**
			Other	4	0.40	0.18 to 0.63
	Provider	19.33 (<.001)	Self-delivered	3	0.39	-0.39 to 1.17
			Therapist	14	0.11	0.29 to 0.59
			**Guided**	**4**	**0.85**	**0.63 to 1.08**
	Delivery	20.56 (<.001)	Face-to-face	13	0.43	0.27 to 0.59
			Online	4	0.39	-0.02 to 0.80
			**Combined**	**4**	**0.85**	**0.63 to 1.08**
	Format	20.28 (<.001)	Individual	3	0.39	-0.02 to 0.80
			Group	15	0.44	0.31 to 0.58
			**Combined**	**4**	**0.85**	**0.63 to 1.08**
Between-groups anxiety	Comparison	4.56 (.03)	**Inactive**	**12**	**0.49**	**0.33 to 0.64**
			Active	9	0.27	0.09 to 0.44
	Risk of bias	84.92 (<.001)	Serious risk	2	0.34	-70 to 1.37
			High risk	1	-0.08	-0.42 to 2.53
			**Some concerns**	**4**	**1.24**	**0.98 to 1.49**
Within-subject anxiety	Intervention	15.99 (.001)	**Mindful+Comp.**	**16**	**0.56**	**0.41 to 0.72**
			MBRS	3	0.19	-0.07 to 0.45
			MBCT	1	0.27	0.21 to 0.51
			Other	7	0.36	-0.03 to 0.57
	Provider	13.46 (.001)	**Self-delivered**	**6**	**0.50**	**0.27 to 0.74**
			Therapist	18	0.33	0.23 to 0.43
			Guided	2	0.95	-1.29 to 3.21
	Delivery	13.80 (.001)	Face-to-face	17	0.32	0.22 to 0.41
			**Online**	**7**	**0.46**	**0.29 to 0.64**
			Combined	2	0.95	-1.29 to 3.21
	Format	13.99 (.001)	**Individual**	**6**	**0.50**	**0.27 to 0.74**
			Group	19	0.32	0.23 to 0.42

Outcome	Variable	*Q* test (*p* value)	Subgroup	k	SMD	95% CI
			Combined	2	0.95	-1.29 to 3.21
	Risk of bias	28.11 (<.001)	Poor	3	0.37	-0.41 to 1.14
			Serious risk	5	0.20	0.10 to 0.29
			High risk	5	0.48	0.27 to 0.70
			Fair	3	0.36	-0.14 to 0.86
			**Some concerns**	**11**	**0.61**	**0.41 to 0.80**
Between-groups depression	Intervention	8.06 (.04)	**Mindful+Comp.**	**13**	**0.48**	**0.17 to 0.78**
			MBRS	2	0.22	-0.49 to 0.94
			MBCT	1	0.19	-0.20 to 0.59
			Other	3	0.48	0.11 to 0.85
	Provider	35.30 (<.001)	Self-delivered	2	-0.14	-0.57 to 0.29
			**Therapist**	**16**	**0.48**	**0.27 to 0.70**
	Format	35.30 (<.001)	Individual	2	-0.14	-0.57
			**Group**	**17**	**0.47**	**0.27 to 0.67**
Within-subject depression	Risk of bias	20.46 (<.01)	Poor	4	0.21	-0.01 to 0.43
			High risk	7	0.44	-0.04 to 0.93
			Fair	1	0.17	-0.02 to 0.37
			Serious risk	3	0.25	0.06 to 0.44
			**Some concerns**	**9**	**0.50**	**0.39 to 0.62**
	Risk of bias	18.79 (<.001)	Serious risk	2	0.15	-0.62 to 0.92
			**Some concerns**	**1**	**1.48**	**0.89 to 2.08**
			High risk	2	0.25	-0.90 to 1.41
Between-groups mindfulness	Provider	31.83 (<.001)	Self-delivered	5	0.17	0.02 to 0.32
			**Therapist**	**21**	**0.62**	**0.46 to 0.78**
			Guided	2	0.84	-1.28 to 2.96
	Delivery	7.81 (.02)	Online	7	0.31	0.02 to 0.62
			**Face-to-face**	**21**	**0.62**	**0.46 to 0.78**
			Combined	2	0.84	-1.28 to 2.96
	Format	34.94 (<.001)	Individual	5	0.17	0.02 to 0.32
			**Group**	**23**	**0.63**	**0.48 to 0.78**
			Combined	2	0.84	-1.28 to 2.96
Within-subject mindfulness	Provider	7.77 (.02)	Self-delivered	6	0.34	0.18 to 0.50
			Therapist	26	0.51	0.38 to 0.64
			**Guided**	**4**	**0.95**	**0.14 to 0.50**
	Format	7.95 (.02)	Individual	6	0.34	0.18 to 0.50
			Group	28	0.51	0.39 to 0.63
			**Combined**	**4**	**0.95**	**0.14 to 0.50**

Outcome	Variable	*Q* test (*p* value)	Subgroup	k	SMD	95% CI
Between-groups self-compassion	Provider	11.94 (.01)	**Therapist**	**23**	**0.65**	**0.41 to 0.89**
			Self-delivered	4	0.02	-1.13 to 1.18
			Guided	2	1.01	-0.02 to 2.05
	Delivery	13.80 (.001)	**Face-to-face**	**22**	**0.58**	**0.37 to 0.79**
			Online	6	0.48	-0.67 to 1.63
			Combined	2	1.01	-0.02 to 2.05
	Format	12.32 (.001)	**Group**	**24**	**0.65**	**0.42 to 0.89**
			Individual	4	0.02	-1.13 to 1.18
			Combined	2	1.01	-0.02 to 2.05
Within-subject self-compassion	Diagnosis	6.85 (.001)	No diagnosis	40	0.58	0.42 to 0.73
			**Axis I**	**3**	**1.11**	**0.29 to 1.93**
	Intervention	22.32 (<.001)	Mindful+Comp.	24	0.70	0.46 to 0.95
			MBRS	5	0.12	-0.13 to 0.37
			**MBCT**	**4**	**0.85**	**0.18 to 1.52**
			Other	10	0.49	0.37 to 0.62
	Risk of bias	12.22 (.01)	Serious risk	7	0.31	0.06 to 0.56
			High risk	11	0.49	0.15 to 0.74
			Fair	4	0.62	0.19 to 1.05
			**Some concerns**	**15**	**0.85**	**0.58 to 1.11**
			Poor	6	0.69	-0.11 to 1.49

Note. SMD = standardized means difference; CI = confidence intervals
